# A new nucleosomic-based model to identify and diagnose SSc-ILD

**DOI:** 10.1186/s13148-020-00915-4

**Published:** 2020-08-17

**Authors:** Julien Guiot, Monique Henket, Béatrice Andre, Marielle Herzog, Nathalie Hardat, Makon-Sebastien Njock, Catherine Moermans, Michel Malaise, Renaud Louis

**Affiliations:** 1 Pneumology Department, CHU Liège, Domaine Universitaire du Sart-Tilman, B35, B4000, Liège, Belgium; 2 Rheumatology Department, CHU Liège, Domaine Universitaire du Sart-Tilman, B35, B4000, Liège, Belgium; 3 Belgian Volition SPRL, Parc Scientifique Créalys, 22 rue Phocas lejeune, B5032 Isnes, Belgium

## Abstract

**Background:**

Systemic sclerosis (SSc) is a rare connective tissue disease associated with rapid evolving interstitial lung disease (SSc-ILD), driving its mortality. Specific biomarkers associated with the evolution of the lung disease are highly needed. We aimed to identify specific biomarkers of SSc-ILD to predict the evolution of the disease. Nucleosomes are stable DNA/protein complexes that are shed into the blood stream making them ideal candidates for biomarkers.

**Methods:**

We studied circulating cell-free nucleosomes (cf-nucleosomes) in SSc patients, 31 with ILD (SSc-ILD) and 67 without ILD. We analyzed plasma levels for cf-nucleosomes and investigated whether global circulating nucleosome levels in association with or without other biomarkers of interest for systemic sclerosis or lung fibrosis (e.g., serum growth factors: IGFBP-1 and the MMP enzyme: MMP-9), could be suitable potential biomarkers for the correct identification of SSc-ILD disease.

**Results:**

We found that H3.1 nucleosome levels were significantly higher in patients with SSc-ILD compared SSc patients without ILD (*p* < 0.05) and levels of MMP-9 were significantly increased in patients with SSc-ILD compared to SSc patients without ILD (*p* < 0.05). Conversely, IGFBP-1 was significantly reduced in patients with SSc-ILD compared to SSc without ILD (*p* < 0.001). The combination of cf-nucleosomes H3.1 coupled to MMP-9 and IGFBP-1 increased the sensitivity for the differential detection of SSc-ILD. High levels of accuracy were reached with this combined model: its performances are strong with 68.4% of positive predictive value and 77.2% of negative predictive value for 90% of specificity. With our model, we identified a significant negative correlation with FVC % pred (*r* = −0.22) and TLC % pred (*r* = −0.31). The value of our model at T1 (baseline) has a predictive power over the Rodnan score at T2 (after 6-18 months), showed by a significant linear regression with *R*^2^ = 19% (*p* = 0.013). We identified in the sole group of SSc-ILD patients a significant linear regression with a *R*^2^ = 54.4% with the variation of DLCO between T1 and T2 (*p* < 0.05).

**Conclusion:**

In our study, we identified a new blood-based model with nucleosomic biomarker in order to diagnose SSc-ILD in a SSc cohort. This model is correlated with TLC and FVC at baseline and predictive of the skin evolution and the DLCO. Further longitudinal exploration studies should be performed in order to evaluate the potential of such diagnostic and predictive model.

## Introduction

Systemic sclerosis (Ssc) is a rare inflammatory disease of unknown origin associated with multi-organic involvement [1]. The main complication of SSc driving the morbi-mortality of the disease is the particular appearance of interstitial lung disease (ILD) [2]. The clinical history of SSc associated interstitial lung disease (SSc-ILD) can vary from a slow evolving lung disease to a quick flare up and deterioration. Treatment is based on aggressive immunosuppression only proposed in cases of progressive lung disease. Nowadays, in the more severe cases, an autologous hematopoietic stem cell transplantation can be recommended [3]. The problem, as in other interstitial lung diseases [4, 5], is to identify patients at increased risk of progression for early therapeutic intervention.

SSc is typically associated with vascular inflammation and fibrosis [6]. Its clinical classification is based on skin fibrosis extension separating patients in two different patterns: Limited cutaneous systemic sclerosis (lcSSc) characterized by a skin fibrosis restricted to distal areas to the elbows and knees. The second one is the diffuse cutaneous systemic sclerosis, which is associated with the involvement of proximal areas, the face, and the trunk in addition to distal areas.

Biological markers, often referred as biomarkers, are commonly defined as objectively measured elevated indicators of physiological/pathological processes or pharmacological response to therapeutic intervention [4].

Biomarkers remain urgently needed as tools for differential diagnosis, prognosis, and disease progression and as therapeutic response predictors. Although anti-nuclear antibodies (ANA) were the first biomarkers available in Sscl, they still are unhelpful for the assessment of disease activity and treatment response.

Nucleosomes are the basic unit of chromatin consisting in a 147 bp DNA strand wrapped around a protein octamer of core histones (H2A, H2B, H3, and H4). Nucleosomes are released from dying or stressed cells into blood circulation. Elevated nucleosome levels are often found in various cancer and in acute and chronic non-malignant inflammatory diseases [7, 8]. Even if elevated levels of circulating cell-free nucleosomes (cf-nucleosomes) does not appear to be specific to a unique pathological state and correlate with different mechanisms of release, it may be of a particular interest for differential diagnosis and may serve as a guiding biomarker for prognosis in association with other biomarkers as it is sensitive and linked with inflammatory processes [9–12]. Reinforcing this hypothesis, Yoshizaki et al. identified a significant increase in cell-free nucleosomic biomarkers in SSc with a specific correlation with the skin involvement [13].

In this study, we investigated whether there was an association between the level of circulating H3.1 containing nucleosomes and other biomarkers of interest in systemic sclerosis or lung fibrosis such as the serum growth factors IGFBP-1 and the MMP enzyme MMP9, as a suitable approach or the correct identification of SSc-ILD disease [14–19].

## Methods

### Subject characteristics

We selected 98 samples from patients with SSc initially recruited for a previous study (2010 to 2018) focusing on biomarkers in SSc from our ambulatory care policlinic at CHU Liege. The patients were divided into 2 groups: SSc patients without any lung involvement (SSc, *n* = 67) and patients suffering from SSc associated with interstitial lung disease (SSc-ILD, *n* = 31). The diagnosis of SSc was made according to the international recommendations of ACR/Eular [20, 21]. Patients were divided in two groups according to their HRCT scan to determine the presence of ILD. Lung involvement was evaluated using the respiratory function test, HRCT scan, bronchoalveolar lavage (when available), and the clinical history of the patient. We excluded all other causes of interstitial lung disease (such as asbestosis, idiopathic pulmonary fibrosis, hypersensitivity pneumonitis, or toxic pneumonitis). Then, we compared the baseline level of biomarkers (T1) with the time 2 (T2) level of biomarkers collected between 6 and 18 months after T1 (median: 11 months and 22 days).

The protocol was approved by the ethics committee of CHU of Liège, and all subjects gave written consent for their enrollment (Belgian number: B707201422832; ref: 2014/302).

### Pulmonary function tests

We performed lung function tests in our routine respiratory laboratory of CHU Liège. All spirometric tests performed for this study were measured using the pneumotachograph Jaeger Master lab system (Erich Jaeger GmbH, Wuzburg, Germany). The forced expiratory volume in one second (FEV1) and forced vital capacity (FVC) were measured in accordance with the recommendations of the European Respiratory Society (ERS) [22]. The results were expressed in milliliter and percent predicted. The Tiffeneau index or FEV1/FVC was expressed in percent. The total lung capacity (TLC) was measured by body plethysmography according to ERS recommendations (Erich Jaeger GmbH, Wuzburg, Germany). The diffusion capacity of CO (DLCO) and the report DLCO/AV (alveolar volume) were measured by the single-breath carbon monoxide gas transfer method and expressed as percent predicted (SensorMedics2400He/CO Analyzer System, Bilthoven, Netherlands).

### Biomarkers measurements in serum

Level of IGFBP-1 was assessed by ELISA multiplex using Fluorokine-1 as referred to our previous study [18]. The detection limit for this assay was 170 pg/ml. Likewise, the concentration of MMP-9 was analyzed with DuoSet kit (R&D systems). The lower detection limit for this kit was 25 pg/ml. Levels of blood biomarkers were evaluated at baseline (T1) and after 6-18 months (T2).

### Biomarker measurements in plasma

Cell-free nucleosome levels were measured using Nu.Q™ H3.1 ELISA kit (Belgian Volition SPRL, Isnes, Belgium) according to the manufacturer’s instructions. In brief, plasma samples (20 μl in duplicate) were incubated for 2 h 30 min at room temperature in a 96-well microtiter plate coated with a monoclonal antibody raised again a Histone H3.1 epitope. After washing steps, the level of cf-nucleosomes was quantified by adding a biotinylated anti-nucleosome detection antibody directed to a nucleosome conformational epitope (incubation 90 min at room temperature). The wells were washed and streptavidin-horseradish peroxidase (HRP) was added. After incubation for 30 min at room temperature, the wells were washed and a peroxidase substrate-2,2′-azino-bis-(3-ethylbenzothiazolonine-6 sulfonic acid) was added. The optical densities of the wells were read with a Spectramax ID5 microplate reader (Molecular Devices).

### Statistical analysis

All statistics were computed with the software IBM SPSS Statistics, version 26.0.0.0. The majority of the tests performed were nonparametric, using binomial distribution and natural logarithms (logistic regression), mean ranks, medians, and/or interquartile ranges. Cumulative performances of plasma cf-nucleosomes and serum biomarkers were evaluated using multivariate predictive analysis. For this, models were developed with Binary Logistic Regression (stepwise/backward method; Wald and chi-square statistics), providing the most discriminant single biomarkers or combinations of biomarkers, and giving back probabilities of suffering from SSc-ILD in relation to SSc for each patient and each model computed. XY plots were done to express the results in terms of real data, with an estimation of each biomarker/model’s predictive power on respiratory function levels using simple linear regression and FIT curves (prediction lines).

## Results

### Subject demographic and functional characteristics

The demographic, functional, and treatment characteristics of the subjects are given in Table 1. Whereas the mean age of patient is similar between SSc and SSc-ILD, SSC-ILD exhibit a lower TLC and FVC than those without ILD (*p* < 0.001 and *p* < 0.01 respectively) with a reduced DLCO compared to those with preserved lung parenchyma (*p* < 0.01).

**Table 1:** Subjects characteristics

	SSc (*n* = 67)	SSc-ILD (*n* = 31)
**Age, years**	56 ± 12	60 ± 13
**Gender (M/F)**	17/50	6/25
**BMI, kg/m**^**2**^	25 ± 4	25 ± 4
**Smokers (NS/FS/S) (%)**	48/28/24	55/28/17
**Paq-year**	15 ± 17	6.9 ± 8.4
**CRP**	1.7 (0.7-5.1)	2.4 (1.1-6.1)
**FEV1 post-BD, % pred**	98 ± 22	88 ± 23*
**FVC post-BD, % pred**	104 ± 20	90 ± 22**
**FEV1/FVC post-BD, % pred**	78 ± 10	81 ± 8
**TLC, % pred**	101 ± 15	85 ± 19***
**TLCo % pred**	71 ± 20	56 ± 16**
**KCO % pred**	79 ± 19	74 ± 14
**HRT extent (% lung)**	/	5 (5-25)
**IT, immunosupressive therapy (%)**	14	38
**OCS, oral cortico steroide (%)**	24	28
**Prednisolone equivalent (mg)**	5 (5-5)	10 (5-10)
**GI tract score, none/mild/severe (%)**^**1**^	36/62/2	16/80/4
**Disease duration (year)**	6.93 ± 8.47	5.55 ± 5.87
**Rodnan skin score**	2 (0-5)°	2 (0-4)°
**limited SSc/lcSSc/dSSc/sine scleroderma (%)**	38/59/6.3/4.8	41/41/14/0
**Musculoskeletal involvement (%)**	22	21
**Renal crisis (%)**	6.8	0
**Cardiac involvement (%)**	2	37

Data are expressed as mean ± SD

*NS* non-smoker, *FS* former smoker, *S* smoker, *IT* immunosuppressive therapy (mycophenolate mofetil, methotrexate, cyclophosphamide), *SSc* systemic sclerosis, *lcSSc* limited cutaneous, *dSSc* diffuse cutaneous SSc, *SS* sine scleroderma

°Values are expressed as mean ± SD when parametrics and median (IQR) when non parametrics

^1^GI tract score, missing value: SSc 20/67; SSc-ILD 12/31

*p < 0.05

**p < 0.01

***p < 0.001 compared to healthy subjects

### Circulating cell-free nucleosome levels in SSc and SSc-ILD

**Fig. 1:**
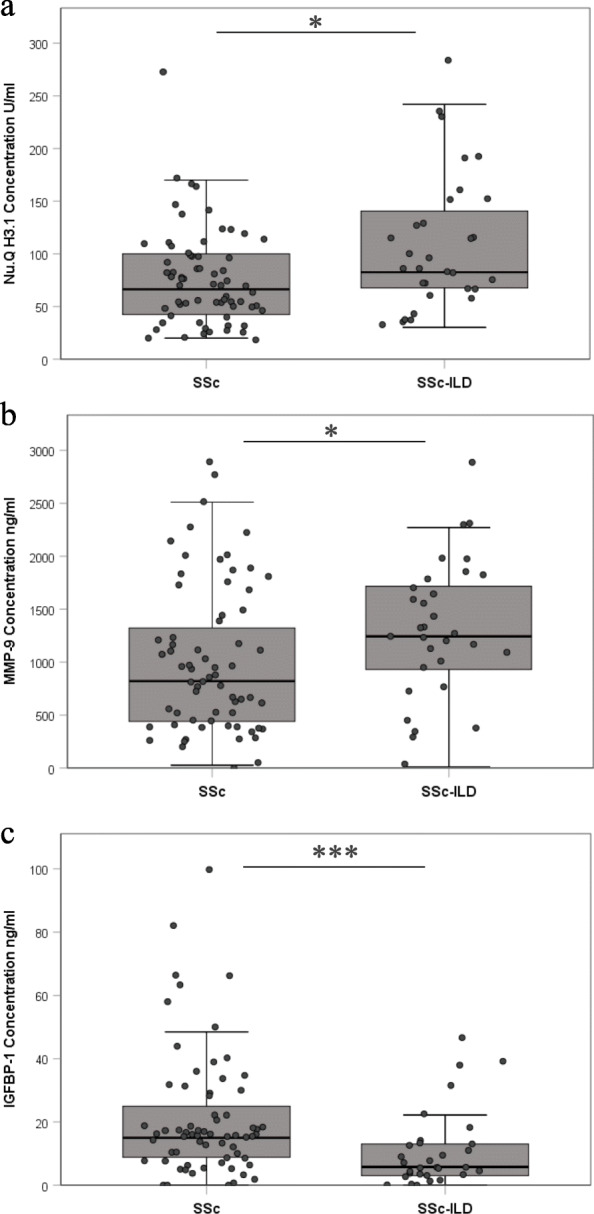
Individual biomarkers in the blood of SSc compared to SSc-ILD at baseline. Boxplot expressing medians and IQ range for each biomarker in function of diagnosis at T1. The box plots showed significantly higher levels for H3.1 containing cf-nucleosomes (**a**) and MMP-9 (**b**) in patients with SSc-ILD (*n* = 31) compared with SSc patients (*n* = 67) (*p* < 0.05 for both), in contrast IGFBP-1 (**c**) was significantly lower level in patient with SSc-ILD (*p* < 0.001). *P* values were determined by Mann-Whitney rank-sum test. The box plot shows the median and the 25th and 75th percentiles; the whiskers indicate 1.5 times the interquartile range (IQR)

We found that the baseline H3.1 nucleosome levels were significantly higher in patients with SSc-ILD compared to patients with SSc without ILD (Table 2; Fig. 1a; *p* < 0.05). The area under the ROC curve (AUC) for cf-nucleosomes H3.1 was 0.68 for the discrimination of SSc-ILD vs. SSc group (Supplementary Figure 1).

**Table 2:** Levels blood biomarkers

Biomarkers	T1 (*n* = 98)					T2 (*n* = 37)				
	SSc (*n* = 67)		SSc-ILD (*n* = 31)		Sig.	SSc (*n* = 27)		SSc-ILD (*n* = 10)		Sig.
	Median	IQR	Median	IQR		Median	IQR	Median	IQR	
MMP-9	820.5	435-1354	1243.4	887-1724	*	1164.9	389-1599	903.14	366-1647	
IGFBP-1	14.96	8.6-25.5	5.74	2.2-14.3	***	11.04	7.3-24.6	5.6	2.7-33.1	
H3.1	66.4	41-102	82.6	65-146	*	56.2	41-79	77.6	55-172	*

The levels of IGFBP-1, MMP-9, and H3.1 containing cf-nucleosomes (median and IQR) were evaluated at T1 and T2 for SSc and SSC-ILD patients. Significant differences were observed between SSc and SSc-ILD patients for all three biomarkers at T1. At T2, only the level of H3.1 containing cf-nucleosome remained significantly higher in SSc-ILD patients

*P* values were determined by Mann-Whitney rank-sum test

### Protein biomarkers

We identified that serum levels of MMP-9 were significantly increased in SSc-ILD compared to SSc patients (*p* < 0.05) (Table 2; Fig. 1b). Conversely, IGFBP-1 was significantly reduced in SSc-ILD compared to SSc (*p* < 0.001) (Table 2; Fig. 1c).

### Cumulative performance of cf-nucleosomes and serum biomarkers

**Fig. 2:**
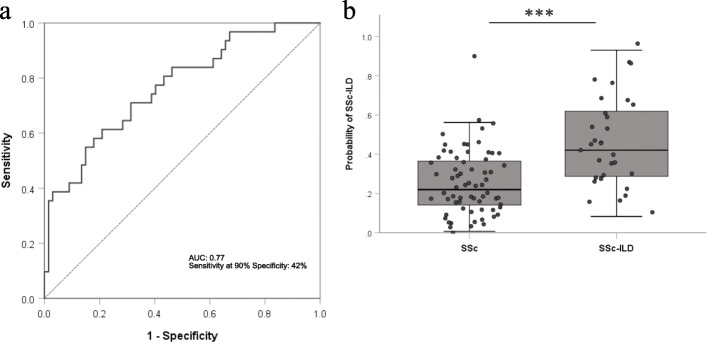
Roc curve and box plot for discrimination of SSc vs SSc-ILD in the combination of H3.1 containing cf-nucleosome associated with IGFBP-1 and MMP-9. A model with three biomarkers: H3.1 containing cf-nucleosome, MMP-9, and IGFBP-1 discriminated patients with scleroderma with fibrosis (SSc-ILD) versus scleroderma without fibrosis (SSc): **a** ROC curves for discrimination of SSc-ILD patients vs SSc patients. The model reached a sensitivity of 58% and 42% at respectively 80% specificity and 90% specificity. The AUC was 0.77 (*p*_Model_ < 0.001; *p*_Nu.Q H3.1_ = 0.022; *p*_MMP-9_ = 0.047; *p*_IGFBP-1_ = 0.01). **b** Box plot demonstrating significantly higher score in patients with a SSC-ILD (*n* = 31) compared with SSc patients (*n* = 67) (*p* < 0.001). The score for each group was achieved with pre-processed ELISA data from Nu.Q™ H3.1, MMP-9, and IGFBP-1 assays. A binary logistic regression model was used to calculate the probability of SSc-ILD in relation to SSc. *P* values were determined by Mann-Whitney rank-sum test. The box plot shows the median and the 25th and 75th percentiles; the whiskers indicate 1.5 times the interquartile range (IQR)

The best model was found by combining H3.1 containing cf-nucleosome and MMP-9 and IGFBP-1 levels. It increased the sensitivity for the differential detection of the SSc-ILD to 42% at 90% specificity with an area under the curve of 0.77 compared with the best single assay sensitivity at 32% observed with the H3.1 containing cf-nucleosome (Fig. 2a and Table S2). Box plot showed a significant higher score in patients with SSc-ILD than SSc patients without ILD (Fig. 2b, *p* < 0.001).

High levels of accuracy were reached with this combined model: with 68.4% positive predictive value and 77.2% negative predictive value for at 90% specificity.

We also tested a model including the gender as a supplementary explanatory variable in the model combining the three biomarkers to explain SSc-ILD in relation to SSc (Supplementary Figure 2). Gender is not significant (*p* = 0.269) in this new model, and the prediction power is not statistically improved (*R*^2^ = 29.4%) (data not shown). However, the gender seems having an impact on the levels of the combined model with Nu.Q H3.1, IGFBP-1, and MMP-9 only in the SSc-ILD group: a two-way ANOVA showed an interaction between the gender and the disease (*p* = 0.027) on the probability of SSc-ILD provided by the model (Supplementary Figure 2). The probability of SSc-ILD is higher for males than for females in the SSc-ILD group. Nevertheless, the interpretations of these results should be cautious because of the great difference of sizes between groups (SSc group: 50 females and 17 males; SSc-ILD group: 25 females and 6 males).

In addition, models with only two out of the three biomarkers were also evaluated (Supplementary Figure 3). Nevertheless, by comparing the area under the curve, the sensitivity at 90% specificity, and the coefficient of determination *R*^2^ of these models, none of them was better than the model combining H3.1 containing cf-nucleosome and MMP-9 and IGFBP-1 levels.

### Correlation between biomarkers and pulmonary function tests

Correlations between H3.1 containing cf-nucleosomes, IGFBP-1, and MMP-9 in the global cohort compared to PFTs are represented in the supplementary material (Table S1). At baseline level, IGFBP-1 was positively correlated with TLC (% pred) (*r* = 0.267) and negatively correlated with KCO (DLCO/VA) (*r* = −0.247). We did not find any correlation for MMP-9.

For cf-nucleosome, we identified a negative correlation with DLCO (*r* = −0.272) and a positive correlation with Rodnan skin score (*r* = 0.268) [23]. At T2, H3.1 containing cf-nucleosome levels was negatively correlated with TLC (% pred) (*r* = −0.413) and positively correlated with Rodnan score (*r* = 0.454). Of note, we did not find any correlation between the two other biomarkers and PFT at T2.

### Predictive value of the model

**Fig. 3:**
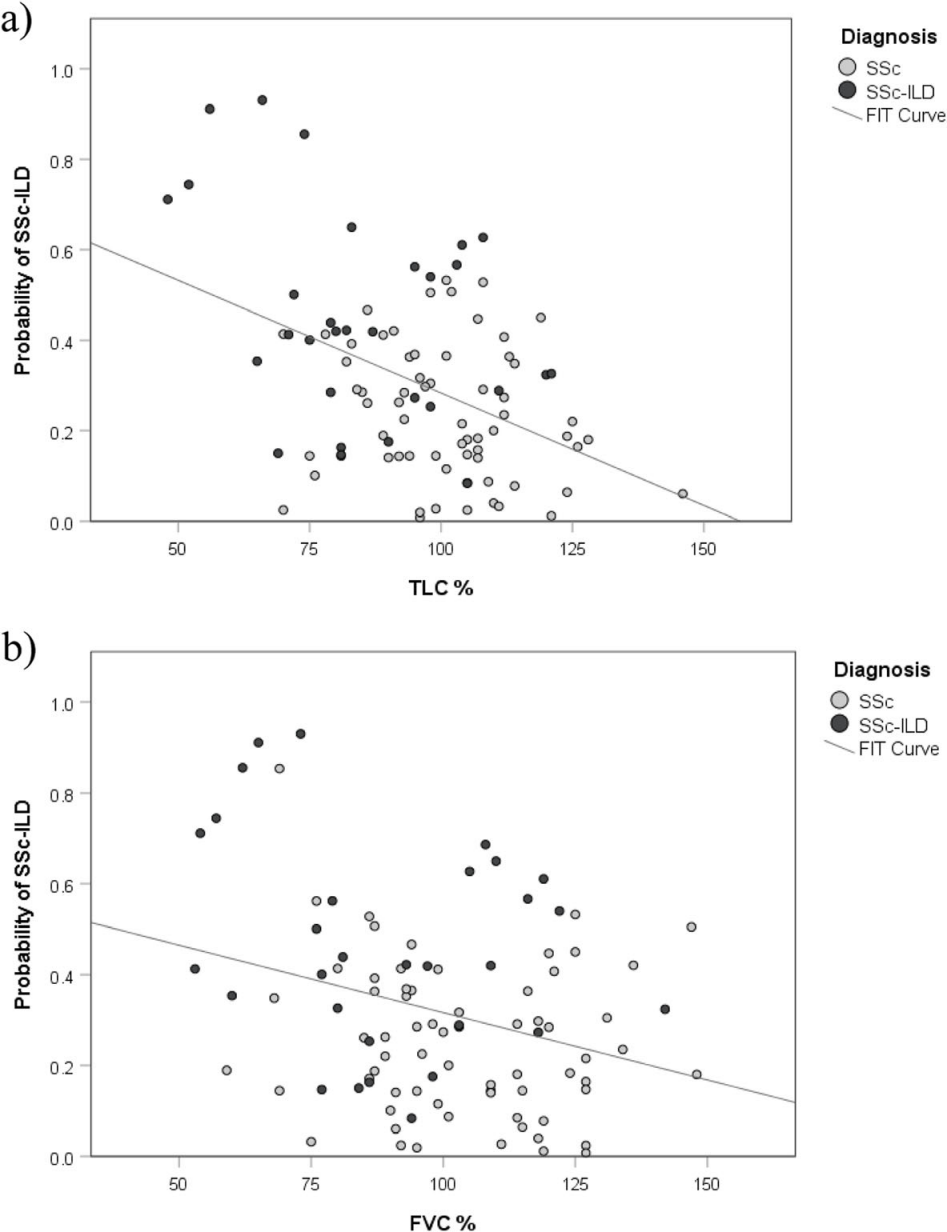
Correlation of the combined model IGFBP-1, MMP-9, and H3.1 containing cf-nucleosomes against FVC (% pred) and TLC (% pred). Significant negative correlations are observed with FVC (*r* = −0.218, *p* < 0.01) and with TLC (*r* = −0.315, *p* < 0.05)

We identified with our combination of three biomarkers a significant correlation with FVC (% pred) and TLC (% pred) (Fig. 3). Our model was negatively correlated with FVC (*r* = −0.218) and with TLC (*r* = −0.315). At T2, the model is even more negatively correlated with TLC (% pred) (*r* = −0.431) but not with other parameters (Table 3). When split by groups (SSc or SSc-ILD), the highest significant correlations are found for SSc-ILD patients: the model is negatively correlated with TCL (% pred) (*r* = −0.576).

**Table 3:**
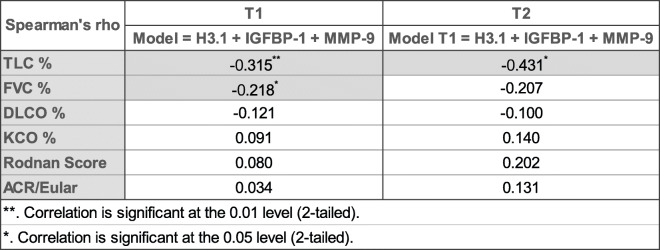
Correlations between the model and PFT at baseline

The level of the combined model with IGFBP-1, MMP-9, and H3.1 containing cf-nucleosomes (probability of SSC-ILD) computed at T1 was evaluated in relation to PFT for all patients. Significant negative correlations are observed between the model and FVC (% pred) and TLC (% pred). The negative correlation is still significant and even stronger between the model computed with T1 measurements and TLC (% pred) quantified at T2 (but not with FVC at T2), which means that the model could be a predictor of disease progression for TLC (% pred) at T2

**Fig. 4:**
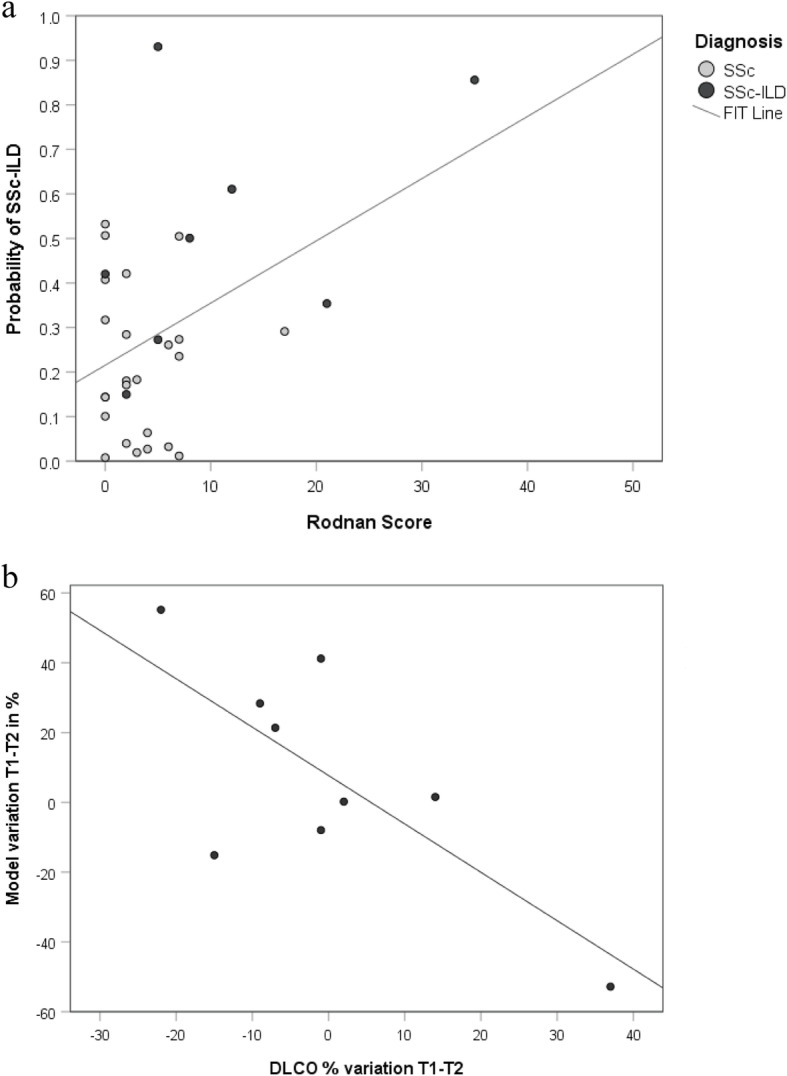
Predictive value of the model. Linear regression with the model as an explanatory variable for PFT. **a** A significant linear regression with all patients (SSc + SSc-ILD), *p* = 0.013, with *R*^2^ = 19%; the score of the model H3.1 containing cf-nucleosome, IGFBP-1, MMP-9 at T1 has a predictive power over the Rodnan score at T2 (*n* = 32, *n*_SSc_ = 24, and *n*_SSc-ILD_ = 8). **b** A Significant linear regression with *p* = 0.023 and *R*^2^ = 54.4% was observed for the SSc-ILD group: variations of DLCO between T1 and T2 is explained by the variation of the model between these two-time points (*n* = 9)

Moreover, the value of our model at T1 has a predictive power over the Rodnan score at T2, showed by a significant linear regression with *R*^2^ = 19% (*p* = 0.013) (Fig. 4a). Then, we evaluated the predictive power of our model over the PFT evolution between T1 and T2 as a potential reliable predictor of the disease prognostic. We identified in the sole group of SSc-ILD patients a significant linear regression with a *R*^2^ = 54.4% with the variation of DLCO between T1 and T2 (*p* < 0.05) (Fig. 4b).

## Discussion

In our study, we identified a new blood-based model combining H3.1 containing cf-nucleosomes, IGBP-1 and MMP-9 as biomarkers to diagnose SSc-ILD in a SSc cohort. This model is correlated with TLC and FVC at baseline and is a consistent predictor of the skin evolution and the DLCO in SSc-ILD patients.

We identified an increased level of circulating H3.1 containing cf-nucleosome in SSc-ILD patients compared to SSc. Of interest, cf-nucleosome H3.1 was negatively correlated with DLCO (% pred) and positively correlated with Rodnan score. Focusing on DLCO analysis, this has been made on a small cohort, which limited the impact of the analysis. Of interest, this observation is in line with a previous study of Yoshizaki et al. who identified a significant increase in cell-free nucleosomic biomarkers in SSc with a specific correlation with the skin involvement [13]. In our study, instead of using total cell-free circulating nucleosomes, we used a specific H3 variant assay (Nu.Q™ H3.1) which seems to increase sensibility compared to our previous study [5]. Contrarily to our previous findings focusing on IPF, we identified an increase in cf-nucleosome levels, which is in keeping with the physiopathology of SSc. Indeed, the lung fibrosing process is associated with lung inflammation recognized in SSc per se. Knowing that we are not surprised that the global level of cf-nucleosome was increased as it is generally described in systemic inflammatory diseases due to the immune activation [8, 24–26]. This observation is in line with the treatment benefit with immunosuppressive therapies shown in SSc-ILD conversely to what is seen in IPF [22].

Nucleosomes may play direct and indirect roles in immunological abnormalities associated with SSc, which could be independent of the antigen-specific pathway [8, 25, 27]. By associating these biomarkers with inflammatory and fibrosis biomarkers, we could expect an increase accuracy to diagnose SSc patients. Indeed, SSc is a highly complex disease with an involvement of multiple pathways like inflammatory and fibrosis process [28].

In our study, we showed that MMP-9 was increased in SSC-ILD compared to SSC patients. Matrix metalloproteinase-9 (MMP-9) has been implicated in the pathogenesis of cancer, autoimmune disease, and various pathologic conditions characterized by excessive fibrosis. Kim et al. suggested that the enhanced production of MMP-9 may contribute to fibrogenic remodeling during the progression of fibrosis in SSc [29] by its action of metalloproteinase. Confirmatory to our findings, MMP-9 is also known to be elevated in BALF of SSc-ILD patients compared to those without ILD [30]. Therefore, MMP-9 could be a specific marker of SSc-ILD activity and would benefit to be specifically studied in one other study.

IGFBP-1 has not been much studied in SSc-ILD so far. Nevertheless, IGF-I is known to be associated with the fibroblastic activity in SSc [31, 32]. The IGFBPs carry IGFs and can increase their half-life, alter their function (in potentiating or inhibiting it), or facilitate their passage to the target tissues [33]. Focusing on those results, we hypothesize that those lower levels of blood IGFBP-1 seen in SSc-ILD patients could increase the potential for IGF activity in the lungs, increasing its unbound form. Conversely to those observations, we identified in our previous study on IPF patients that IGFBP-1 was increased in untreated patients and reduced under anti-fibrotic therapy [18].

IGFBP-1 and MMP-9 were selected to be associated with H3.1 containing cf-nucleosomes because of their correlation with lung disease [34, 35]. By associating those 3 biomarkers, we showed a clear improvement in the potential value of such biomarkers. Our model in that context allowed to discriminate SSc and SSc-ILD with a sensitivity of 58% at 80% of specificity. Of interest, this model is significantly associated with lung volume at baseline and has a predictive power over the lung dysfunction (assessed by DLCO (% pred) at T2).

SSc-ILD is a major comorbidity in SSc patients driving the mortality of the disease. In the context of new proven anti-fibrotic drug therapy (Scensis and Inbuild trial) [36, 37], specific biomarkers are highly needed to identify patients at high risk of progressive lung disease [1]. Therefore, further longitudinal exploratory studies should be performed in order to evaluate the potential of such diagnostic and predictive model.

## Supplementary information

**Additional file 1: Table S1.** Correlation between biomarkers and pulmonary function tests. Correlation of each individual biomarkers: H3.1, IGFBP-1 and MMP-9 at the baseline level of biomarkers T1 and at T2 (between 6 and 18 months after T1) and pulmonary functions tests.

**Additional file 2: Table S2.** Sensitivity at 90% specificity of the individual biomarkers: Nu.Q™ H3.1, IGFBP-1, MMP-9.

**Additional file 3: Supplementary Figure 1.** ROC curve for the discrimination of SSc vs. SSc-ILD. The area under the curve (AUC) reached 0.68 for H3.1 with a diagnostic sensitivity of 32% at 90% specificity.

**Additional file 4: Supplementary Figure 2.** Interaction plot from the Two-Way ANOVA expressing the means for the probability of SSc-ILD provided by the model, by Gender and by Disease severity (p _Disease_ < 0.001 ; p _Gender_ = 0.046 ; p _Disease*Gender_ = 0.027).

**Additional file 5: Supplementary Figure 3.** Roc Curve for discrimination of SSc vs SSc-ILD in models containing a combination of only two biomarkers in comparison with the model combining the three biomarkers H3.1 containing cf-nucleosome associated, IGFBP-1 and MMP-9. The model combining the three biomarkers H3.1 containing cf-nucleosome associated, IGFBP-1 and MMP-9 reached a sensitivity of 42% at 90% Specificity (AUC 0.77; p _Model_ < 0.001 ; p _Nu.Q H3.1_ = 0.022 ; p _MMP-9_ = 0.047 ; p _IGFBP-1_ = 0.01 ; R^2^ = 28%). The model combining the two biomarkers: cf-nucleosome H3.1 and IGFBP-1 reached an AUC of 0.75 and a sensitivity at 90% specificity of 48% (p _Model_ < 0.001 ; p _Nu.Q H3.1_ = 0.010 ; p _IGFBP-1_ = 0.013 ; R^2^ = 23%). The model combining the two biomarkers H3.1 and MMP-9 reached an AUC of 0.69 and a sensitivity at 90% specificity of 32% (p _Model_ = 0.005 ; p _Nu.Q H3.1_ = 0.014 ; p _MMP-9_ = 0.115 ; R^2^ = 14% ). We noted a loss of power with these two models compared to the model combining the three biomarkers. In addition, in the model combining H3.1 and MMP-9, the biomarker MMP-9 isn’t significant (p=0.115) inside the model.

## Data Availability

Data contains information that could be used to identify study participants and is available upon request from the corresponding author at J.Guiot@chu.ulg.ac.be.

## Abbreviations

ANA: Anti-nuclear antibodies
ATS: American Thoracic Society
AUC: Area under the curve
Cf-nucleosomes: Cell-free nucleosomes
CV: Coefficient of variation
DLCO: Diffusion lung capacity for CO
ERS: European Respiratory Society
FEV1: Forced expired volume in one second
FVC: Forced vital capacity
HRCT: High resolution computed tomography
HRP: Streptavidine-horseradish peroxydase
HS: Healthy subjects
IGF: Insulin-like growth factor
IGFBP: Insulin-like growth factor binding protein
ILD: Interstitial lung disease
KCO: Diffusion lung capacity for CO/alvéolar ventilation
lcSSc: Limited cutaneous systemic sclerosis
MMP: Matrix metalloprotéase
ROC: Receiver operating characteristic
SSc: Systemic sclerosis
SSc-ILD: Systemic sclerosis-associated interstitial lung disease
SD: Standard deviation
TLC: Total lung capacity
