# Supplementary material for: A new nucleosomic-based model to identify and diagnose SSc-ILD
Source: Clin Epigenetics. 2020 Aug 17;12:124. doi: 10.1186/s13148-020-00915-4 (PMC7430109; doi:10.1186/s13148-020-00915-4)
Supplement: Supplementary file 2 — Additional file 2: Table S2. Sensitivity at 90% specificity of the individual biomarkers: Nu.Q™ H3.1, IGFBP-1, MMP-9. [file 13148_2020_915_MOESM2_ESM.docx]

| Biomarker | Sensitivity(%) at 90% Specificity |
| --- | --- |
| Nu.q h3.1 | 32.3 |
| IGFBP-1 | 25.8 |
| MMP-9 | 9.7 |
|  |  |
|  |  |
|  |  |
