# Supplementary figures and images for: A new nucleosomic-based model to identify and diagnose SSc-ILD
Source: Clin Epigenetics. 2020 Aug 17;12:124. doi: 10.1186/s13148-020-00915-4 (PMC7430109; doi:10.1186/s13148-020-00915-4)

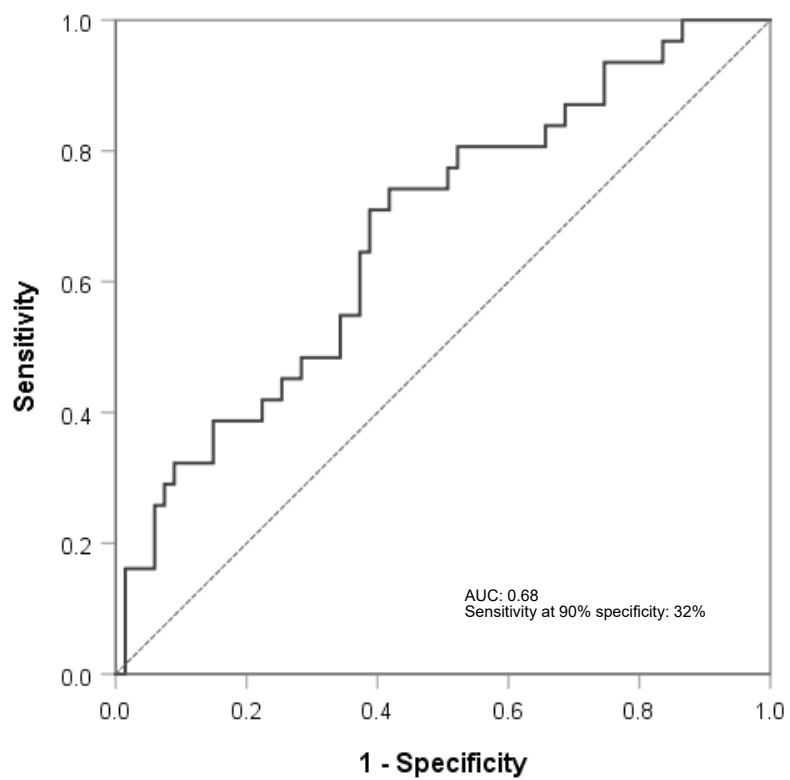

Supplement: Supplementary file 3 — Additional file 3: Supplementary Figure 1. ROC curve for the discrimination of SSc vs. SSc-ILD. The area under the curve (AUC) reached 0.68 for H3.1 with a diagnostic sensitivity of 32% at 90% specificity. [file 13148_2020_915_MOESM3_ESM.pdf]

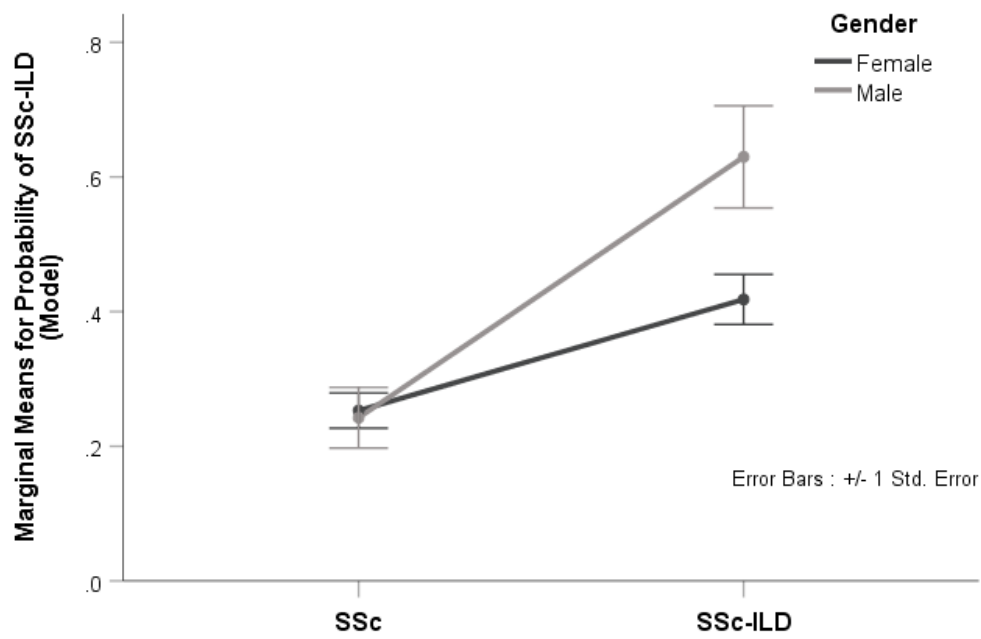

Supplement: Supplementary file 4 — Additional file 4: Supplementary Figure 2. Interaction plot from the Two-Way ANOVA expressing the means for the probability of SSc-ILD provided by the model, by Gender and by Disease severity (p Disease < 0.001 ; p Gender = 0.046 ; p Disease*Gender = 0.027). [file 13148_2020_915_MOESM4_ESM.pdf]

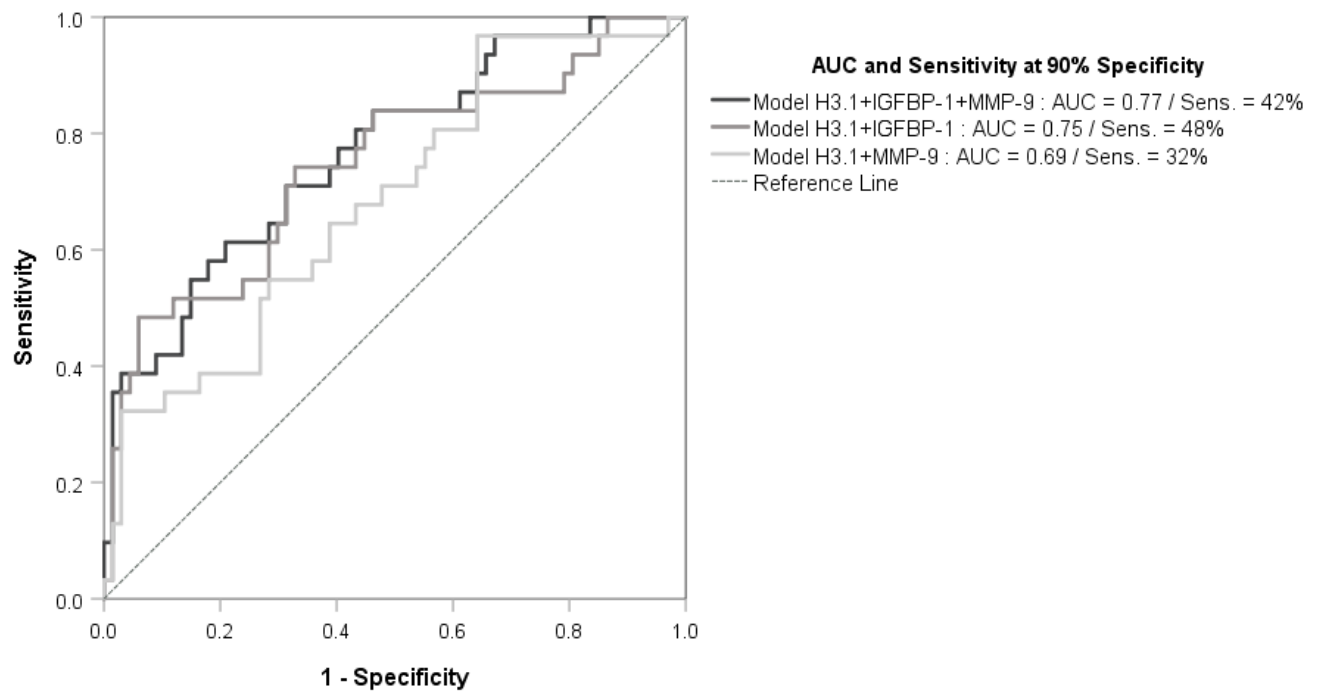

Supplement: Supplementary file 5 — Additional file 5: Supplementary Figure 3. Roc Curve for discrimination of SSc vs SSc-ILD in models containing a combination of only two biomarkers in comparison with the model combining the three biomarkers H3.1 containing cf-nucleosome associated, IGFBP-1 and MMP-9. The model combining the three biomarkers H3.1 containing cf-nucleosome associated, IGFBP-1 and MMP-9 reached a sensitivity of 42% at 90% Specificity (AUC 0.77; p Model < 0.001 ; p Nu.Q H3.1 = 0.022 ; p MMP-9 = 0.047 ; p IGFBP-1 = 0.01 ; R2 = 28%). The model combining the two biomarkers: cf-nucleosome H3.1 and IGFBP-1 reached an AUC of 0.75 and a sensitivity at 90% specificity of 48% (p Model < 0.001 ; p Nu.Q H3.1 = 0.010 ; p IGFBP-1 = 0.013 ; R2 = 23%). The model combining the two biomarkers H3.1 and MMP-9 reached an AUC of 0.69 and a sensitivity at 90% specificity of 32% (p Model = 0.005 ; p Nu.Q H3.1 = 0.014 ; p MMP-9 = 0.115 ; R2 = 14% ). We noted a loss of power with these two models compared to the model combining the three biomarkers. In addition, in the model combining H3.1 and MMP-9, the biomarker MMP-9 isn’t significant (p=0.115) inside the model. [file 13148_2020_915_MOESM5_ESM.pdf]
