# Supplementary material for: A new nucleosomic-based model to identify and diagnose SSc-ILD
Source: Clin Epigenetics. 2020 Aug 17;12:124. doi: 10.1186/s13148-020-00915-4 (PMC7430109; doi:10.1186/s13148-020-00915-4)
Supplement: Supplementary file 3 [file 13148_2020_915_MOESM1_ESM.docx]

Spearman's rho	T1			T2		
	H3.1 U/ml	IGFBP-1 ng/ml	MMP-9 ng/ml	H3.1 U/ml	IGFBP-1 ng/ml	MMP-9 ng/ml
**TLC %**	-0,201	,267**	-0,185	-,413*	0,167	-0,053
**FVC %**	-0,193	0,116	-0,184	-0,127	0,028	-0,220
**DLCO %**	-,272**	-0,020	-0,065	-0,097	-0,169	-0,190
**KCO %**	-0,189	-,247*	0,060	0,163	-0,208	-0,078
**Rodnan Score**	,268*	0,028	-0,027	,454**	-0,157	-0,064
**ACR/Eular**	0,172	0,088	0,195	0,034	-0,214	0,092

**. Correlation is significant at the 0.01 level (2-tailed).

*. Correlation is significant at the 0.05 level (2-tailed).
